# Physicians’ Mutual Benefit Association of Texas

**Published:** 1888-07

**Authors:** 


### PHYSICIANS' MUTUAL BENEFIT ASSOCIATION OF TEXAS.

The members of this Association *must* arouse from their lethargy and indifference. They *must be* stirred up ! But, *what will* move them ? It is too much to expect of the Secretary that he shall send you half dozen notices of assessment, and to have to *dun* you for the contribution of $1.00 levied on each for the beneficiary of a deceased brother. Assessment No. 6, for benefit of the heirs of Dr. Wm. Glover, was sent out April 10, and to date one hundred and twenty-six dollars have been paid in, which amount has been forwarded to Mrs. Glover, (less the expense of collection,) eighty-six dollars at one time and thirty-five dollars lately, and there are still twenty or thirty members who have not responded to notices sent. The names of those who have paid to date are as follows :

Daniel, F. E., Daniels, J. G., Daniel, J. W., Wolff, Sholars, Cupples, Spring, Swearingen, Day, Duchiene, Dupree, J. W., Dupree, W. J., Towsey, Coffey, Buck, Matthews, T. M.; Matthews, W. J., Sterrett, Paine, Brown, Styles, S. F., Styles, T. W., Myers, Powell, Taylor, M. A., Taylor, W. A., Taylor, R. A., Hardy, Weller, McFarland, Inabnit, Tompkins, Paulus, Webb, Harrington, Gray, Ball, Strain, Tidwell, Hill, Morris, Francis, Thhrpe, Throckmorton, Hons. Scales, Perkins, Keating, Osborne, Mayfield, Ford, Murphy, Smith, Q. C., Smith, Bat., Tinsley, Litten, McClintock, Fox, Martin, Gregg, Wilson, Jamison, W. G., Jamison, J. T. Y., Evans, A. D., Evans, A. H., Frazer, Field, Wadgyman, Fennell, Shuford, Burke, Bowers, Patton and Patton, Denman, Talley, Lancaster, Harrison, C. M,, Harrison, R. H., Sr., Harrison, R. H., Jr., Denton, Seymour, Sams, May, Getzwiller, Foster, McGehee, Wakefield, Stinson, Fry, Kaiser, Bell, McCreary, Southworth, Witherspoon, Jones, E.; Bass, J. D.; Musick, Crawford, Bundy, Davis, Hamilton, Burger. Burroughs, Cain, Barbee, Nicholson, Starnes, Abney, Beck, Burrows, Vawter, Robinson, (Todd), Bruce, Maddox, Grace, J. E.; Grace, H. C.; Treadwell, Phillips, Phillips, J. T.; Felder, Reeves, Harmer, Johnson, Burton, Burts.

The twenty odd delinquents have been again notified ; it may be that some failed to receive notices, and whatever amount of the outstanding assessment may be received will be forwarded to Mrs. Glovèr at once.

Assessment No. 7 has been sent out for the benefit of the heirs of Dr. Y. D. Harrington, of Terrell, lately deceased. We hope, members will respond a little more promptly than they did to assessment No. 6.

